# Cutting Periprosthetic Infection Rate: *Staphylococcus aureus* Decolonization as a Mandatory Procedure in Preoperative Knee and Hip Replacement Care—Insights from a Systematic Review and Meta-Analysis of More Than 50,000 Patients

**DOI:** 10.3390/jcm13144197

**Published:** 2024-07-18

**Authors:** Luca Bianco Prevot, Luca Tansini, Accetta Riccardo, Vittorio Bolcato, Livio Pietro Tronconi, Giuseppe Basile

**Affiliations:** 1 Residency Program in Orthopedics and Traumatology, University of Milan, Via Festa del Perdono 7, 20122 Milan, Italy; luca.tansini@unimi.it; 2 Department of Trauma Surgery, IRCCS Ospedale Galeazzi, S. Ambrogio, Via Cristina Belgioioso 173, 20157 Milan, Italy; riccacc@gmail.com (A.R.); basiletraumaforense@gmail.com (G.B.); 3 Astolfi Associati Legal Firm, Via Larga, 8, 20122 Milan, Italy; bolcatovittorio@yahoo.it; 4 Department of Human Sciences, European University of Rome, Via degli Aldobrandeschi 190, 00163 Rome, Italy; ltronconi@gvmnet.it; 5 Maria Cecilia Hospital, Via Corriera, 1, 48033 Cotignola, Italy

**Keywords:** prevention, decolonization, *S. aureus*, THA, TKA

## Abstract

**Background**: No consensus in the literature has been found about the necessity of implementing a decolonization screening protocol for Staphylococcus aureus in patients who undergo prosthesis implantation of the knee (TKA) or of the hip (THA), with the aim of reducing periprosthetic infections (PJIs). **Methods**: A systematic literature search was conducted using PubMed, Web of Science, and Embase in April 2024. Studies conducted on patients who underwent a TKA or THA and who followed a screening and decolonization protocol from *S. aureus* were included. The benefits of implementing this protocol were evaluated through the number of infections overall caused by *S. aureus* and other pathogens. The risk of bias and quality of evidence were assessed using Cochrane guidelines. **Results**: A total of 922 articles were evaluated, and of these, 12 were included in the study for a total of 56,930 patients. The results of the meta-analysis showed a reduced risk of overall PJI (*p* = 0.002), PJI caused by *S. aureus* (*p* < 0.0001), and PJI caused by MRSA (*p* < 0.0001) and highlighted no differences between the two groups in the onset of a PJI caused by other bacteria (*p* = 0.50). **Conclusions**: This study showed that the screening and decolonization of *S. aureus* in patients undergoing THA or THA procedures reduced the risk of a PJI. The screening and decolonization protocol for this kind of patient represents an important procedure for the safety of the patient and in social-economic and medico-legal terms.

## 1. Introduction

Periprosthetic infections (PJIs) after a total knee replacement (TKA) or total hip replacement (THA) implantation represent one of the most frequent and most fearful complications, with important consequences from the point of view of the patient’s quality of life and social impact [1].

The risk of PJI onset after arthroplasty is approximately 2% [2], but this value is on the rise due to the continuous increase in the number of arthroplasties implanted and the increase in the average age and comorbidities of the population undergoing this type of intervention [3,4].

There are several risk factors, both modifiable and non-modifiable, that influence the risk of infection after joint replacement [5].

Among these, there is the colonization of patients by the pathogen Staphylococcus aureus (*S. aureus*), both methicillin-sensitive (MSSA) and methicillin-resistant (MRSA), the greatest concentration of which is reached in the anterior nasal cavity [6].

It has been demonstrated in the literature that patients carrying this bacterium in their commensal flora have an increased risk of infection in a multitude of clinical scenarios, including elective orthopedic surgery [7].

*S. aureus* colonization in the nostrils represents a modifiable risk factor, as preoperative screening and decolonization protocols can be adopted in patients undergoing these elective surgical procedures to potentially reduce infection rates. The clinical effectiveness of these screening/decolonization protocols and their cost/benefit ratio are a topic of debate in the scientific literature as no results have yet been identified that demonstrate superiority in whether or not to perform this preoperative prophylaxis. Some studies have demonstrated decreased rates of periprosthetic joint infection caused by *S. aureus* and increased cost-effectiveness with screening and decolonization [8]. Other studies have demonstrated no change in the rate of PJI caused by MSSA/MRSA when screening and decolonization are applied, but indeed, have shown an increased risk of infection by other pathogens in those patients treated with mupirocin for decolonization [9].

The aim of this work was to evaluate and quantify the advantage of implementing a screening and decolonization protocol in reducing the risk of a PJI caused by *S. aureus* in patients undergoing THA or TKA implantation.

## 2. Materials and Methods

A systematic literature review was performed using the PRISMA (Preferred Reporting Items for Sys. Thematic Reviews and Meta-analysis) guidelines [10].

The systematic review was registered and allocated in the PROSPERO database, National Institute for Health Research, University of York, Center for Reviews and Dissemination (CRD42024557624).

A systematic literature search was performed on 4 April 2024 using PubMed, Web of Science, and Embase using the following string: (staphylococcus aureus OR staphylococcus OR aureus OR MRSA OR MSSA) AND (decolonization OR intranasal administration OR nose OR intranasal OR mupirocin OR chlorhexidine) AND (TKA OR THA OR hip OR hip prosthesis OR replacements OR arthroplasties OR knee prosthesis OR knee).

Duplicates were removed, and subsequently, all records were assessed for suitability based on title and abstract, and, if necessary, the full text was analyzed. 

The inclusion and exclusion criteria are reported in Table 1.

Two independent authors (L.B.P. and L.T.) selected the articles meeting the inclusion criteria; in case of disagreement, this was resolved by the intervention of a third author (R.A.).

### 2.1. Data Extraction

Two independent authors (L.B.P. and L.T.) performed data extraction from the full-text version and supplementary data. Information on the methodology of the study was collected, which included the type of study, the level of evidence, and the year of publication. Patient characteristics were also collected, including the number of patients included and evaluated at follow-up, sex, age, body mass index (BMI), the joint in which the prosthesis was implanted, the type of screening the patients were subjected to, the type of pre-operative antibiotic prophylaxis, the number of infections overall, the number of infections caused by *S. aureus*, and the number of infections caused by other pathogens, the duration of follow-up. Since some data were missing or could not be extrapolated due to the heterogeneity of the clinical studies and the population sample analyzed in the various studies, data was considered missing in the presentation of our results. In cases where the data were only available in graphic format, we proceeded with their extraction using the WebPlotDigitizer tool, as its accuracy in extracting numerical data from graphic data has been demonstrated in previous studies [11,12].

### 2.2. Quality and Risk of Bias Evaluation 

Risk of bias and quality assessments were performed by two separate authors (L.B.P. and L.T.), and discrepancies were resolved through discussion and consensus with a third author (R.A.). The reviewers evaluated the studies considered in the meta-analysis using the Risk Of Bias In Non-randomized Studies of Interventions (ROBINS-I) tool for non-randomized clinical trials, as recommended by Cochrane [13]. The overall quality of evidence for each outcome was graded as high, moderate, low, or very low according to the GRADE (Grading of Recommendations Assessment, Development, and Evaluation) guidelines [14].

### 2.3. Statistic Analysis 

Dichotomous variables were treated using the Mantel–Hanszel test and expressed as the risk ratio (RR). Statistical analysis was performed using the PythonMeta package (version 1.26) in Python (version 3.9). The I2 metric was used to assess heterogeneity and was considered significant when I2  >  25% [15]. Forest plots were used to represent the results of each study and evaluate the aggregate estimates, respectively. In agreement with what was proposed by Borenstein et al. [16], the meta-analysis was performed by implementing a random effects model under the assumption that significant differences between studies could not justify a fixed effects model. When a value of I2 < 25% was found, the meta-analysis was implemented again using a fixed effects model. A *p*-value of 0.05 was set as the significance level for the two-sided test analysis. To calculate the standard deviations not available from the full-text articles, the sample interval was used in accordance with that proposed by Walter and Yao [17].

## 3. Results 

### 3.1. Selection of Articles

**Figure 1:**
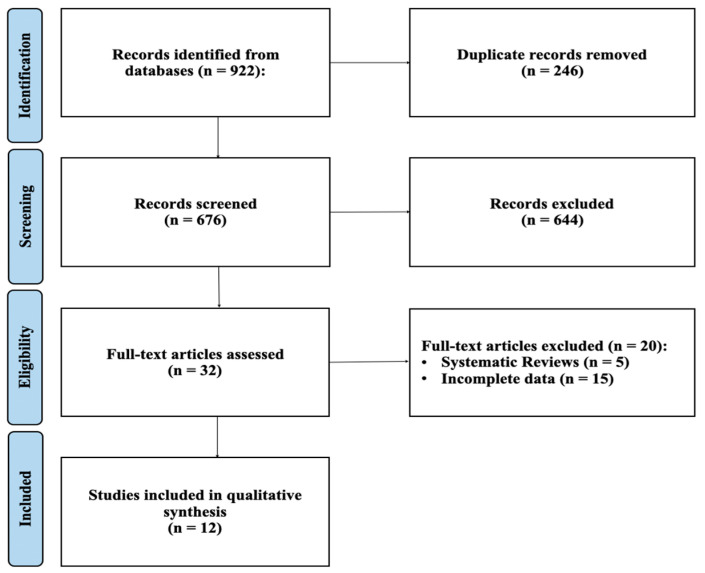
PRISMA flowchart.

The PRISMA article selection flowchart is shown in Figure 1. The literature search produced 270 articles selected from Pubmed, 369 articles from Embase, and 283 articles from Web of Science. Starting from these articles, 246 duplicates were eliminated. Subsequently, 644 articles were eliminated after screening the titles and abstracts. Of the 32 remaining articles, a further 20 articles that did not meet the inclusion criteria were eliminated; at the end of the process, 12 articles remained for the final analysis [18,19,20,21,22,23,24,25,26,27,28,29].

Of the studies analyzed, nine studies were retrospective comparative studies and three studies were prospective observational studies. In total, a population of 56,930 patients undergoing a total hip or knee prosthesis implantation was analyzed, of which 32,382 underwent screening for *S. aureus* colonization and were treated with decolonization protocols, while the other 24,548 underwent surgery without having previously undergone any screening and decolonization protocol.

The sample subjected to screening and decolonization consisted of 57.3% females and 42.7% males and had a mean age of 68 and a standard deviation (SD) of 9.2. A total of 44.8% of the sample underwent total hip replacement surgery, while 55.2% of the sample underwent total knee replacement surgery.

In all included studies, screening was performed via nasal swab, and in two studies, a throat swab was also performed. The screened sample tested positive for MSSA in 21.3% of cases, while 3.4% of patients tested positive for MRSA. The decolonization procedure used in all studies involved the application of intranasal mupirocin two times a day for five consecutive days before surgery (except that of Sankar [28], where povidone iodine or triclosan was also used), while in eight of the studies analyzed, preoperative chlorhexidine body washes were also performed. In all studies, perioperative antibiotic prophylaxis was performed with cefazolin (2 g) associated with vancomycin (1 g) if the patient tested positive for MRSA.

This sample had 0.84% periprosthetic infections, of which 0.22% were supported by MSSA and 0.05% were supported by MRSA.

The control sample, not subjected to screening and decolonization, consisted of 57.1% females and 42.9% males and had an average age of 69 with an SD of 11.6. A total of 42% of the sample underwent total hip replacement surgery, while 58% of the sample underwent total knee replacement surgery.

In all studies, perioperative antibiotic prophylaxis with associated cefazolin (2 g) was performed.

This sample had 1.1% periprosthetic infections, of which 0.5% were supported by MSSA and 0.18% were supported by MRSA. In five studies, it was reported that the diagnosis of periprosthetic joint infection was performed by a positive microbiological culture of periprosthetic fluid/tissue [19,20,23,25,29], while in six studies, the follow-up period was reported, which was at least 1 year [19,23,24,25,26,27]. Full study details are summarized in Table 2.

### 3.2. Meta-Analysis Periprosthetic Joint Infection

**Figure 2:**
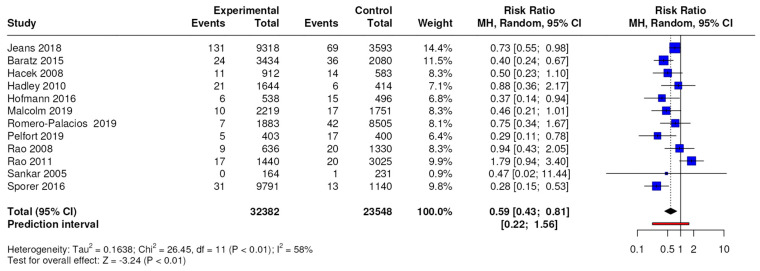
Forest plots for total periprosthetic joint infection; CI = confidence interval, MD = mean difference, *p* = *p*-value. The results of the meta-analysis conducted on 11 studies with a level of evidence of 3 showed differences in terms of the number of MSSA-positive periprosthetic joint infections with an RR of 0.33 (95% CI, 0.23 to 0.47; *p* < 0.0001).

The results of the meta-analysis conducted on 12 studies with a level of evidence of 3 showed significant differences in terms of the number of total periprosthetic joint infections, with an RR of 0.59 (95% CI, 0.43 to 0.81; *p* = 0.002), as shown in Figure 2.

**Figure 3:**
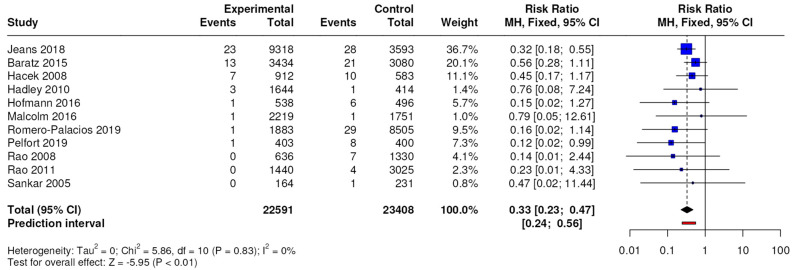
Forest plots for periprosthetic joint infection caused by MSSA; CI = confidence interval, MD = mean difference, *p* = *p*-value.

The results of the meta-analysis conducted on 11 studies with a level of evidence of 3 showed differences in terms of the number of MSSA-positive periprosthetic joint infections, with an RR of 0.33 (95% CI, 0.23 to 0.47; *p* < 0.0001), as shown in Figure 3.

**Figure 4:**
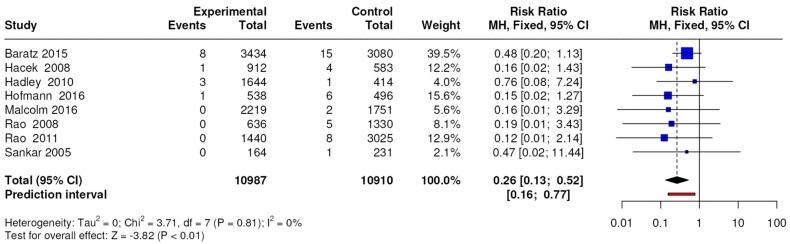
Forest plots for periprosthetic joint infection caused by MRSA; CI = confidence interval, MD = mean difference, *p* = *p*-value.

The results of the meta-analysis conducted on 8 studies with a level of evidence of 3 showed differences in terms of the number of MRSA-positive periprosthetic joint infections, with an RR of 0.26 (95% CI, 0.13 to 0.52; *p* < 0.0001) as shown in Figure 4.

**Figure 5:**
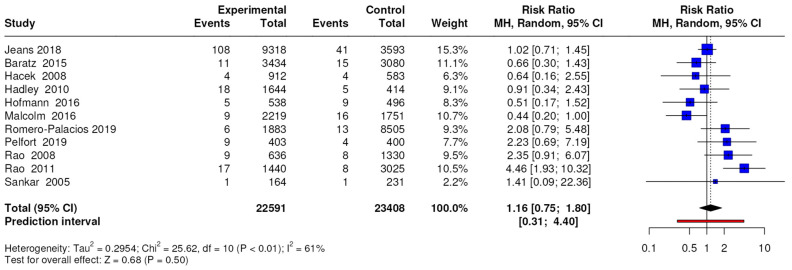
Forest plots for periprosthetic joint infection caused by pathogens other than *S. aureus*; CI = confidence interval, MD = mean difference, *p* = *p*-value.

The results of the meta-analysis conducted on 11 studies with a level of evidence of 3 showed no differences in terms of the number of periprosthetic joint infections caused by pathogens other than *S. aureus,* with an RR of 1.16 (95% CI, 0.75 to 1.80; *p* = 0.50), as shown in Figure 5, while the results of the meta-analysis conducted on three studies with a level of evidence of 3 showed no differences in terms of the number of superficial wound infections, with an RR of 1.29 (95% CI, 0.71 to 2.34; *p* = 0.40).

### 3.3. Risk of Bias and Quality of Evidence

**Figure 6:**
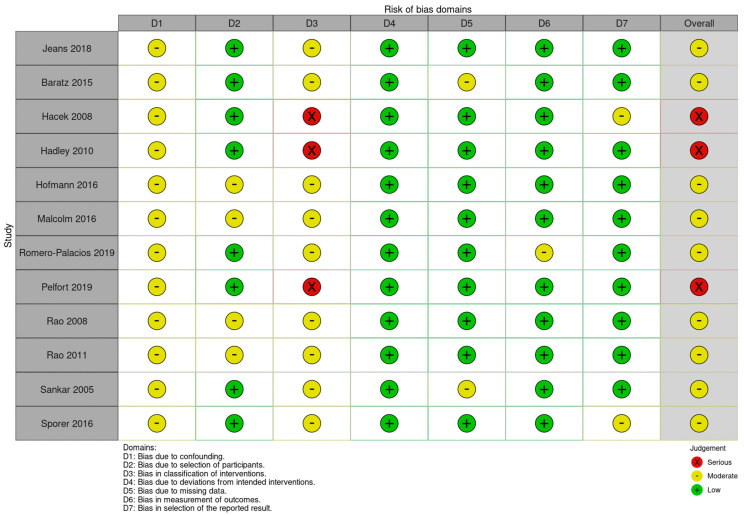
Risk of bias assessments according to the ROBINS tools.

The evaluation using the RoB2 tool showed an overall heterogeneous quality of the studies, with three papers falling in the “Some concerns” category, while the evaluation using the ROBINS-I tool showed an overall heterogeneous quality of the studies, with five papers falling in the “Moderate” category and two papers falling in the “serious” category. Detailed results are shown in Figure 6. 

## 4. Discussion

This systematic review and meta-analysis included 12 independent studies that analyzed 56,930 arthroplasty cases and directly evaluated the effectiveness of decolonization of *S. aureus* in SSI, following primary THA or TKA procedures. Analyses from this study indicated that the screening and decolonization of *S. aureus* reduced the total PJI infections caused by *S. aureus*, resulting in a decrease in infections caused by MRSA; however, no difference was found between the two groups in the onset of PJIs caused by other bacteria. The possible colonization of patients by *S. aureus* who undergo major orthopedic surgery, such as hip or knee prosthesis implantation, is currently of extreme interest. This condition has various implications for clinical practice, in particular from the point of view of the prevention of periprosthetic infections, as it has been demonstrated that colonization by this pathogen significantly increases the risk of periprosthetic infection compared to patients who are not carriers. In fact, in a study by De Buys [30], it is highlighted that colonization with *S. aureus* represents an independent and modifiable risk factor for periprosthetic infections. From the point of view of prevention, this issue is of considerable importance, so much so that in the World Health Organization guidelines for the prevention of surgical site infections, the decolonization of all patients colonized by *S. aureus* is recommended before undergoing operations of major surgery. The nasal cavity is one of the most important sites of colonization by this pathogen. In a study by Chmielowiec-Korzeniowska et al., it emerged that the nasal colonization rate of *S. aureus* in healthy individuals was 20% [31]. A study by Sakr et al. [32] highlights that approximately 30% of the healthy and asymptomatic human population has permanent colonization by *S. aureus*, while a study by Danielli et al. [33] conducted on a population of asymptomatic healthcare workers showed that 42.9% were carriers of *S. aureus*, of which 28.8% were positive for MRSA. Another relevant site that can be colonized by this pathogen is the skin, as demonstrated by various evidence in the literature [34]. These results are of considerable relevance as they allow us to state that colonization by this microorganism represents a non-rare condition and a potential risk that increases the probability of contracting a periprosthetic infection. It is, therefore, necessary to take this aspect into consideration in patients who undergo surgery to implant a hip or knee prosthesis, evaluating the opportunity to implement a preoperative screening and decolonization protocol. The most commonly employed method for the decolonization of *S. aureus* is the topical application of mupirocin twice daily to both nostrils accompanied by or without showers or chlorhexidine skin wipes daily for 5 days prior to surgery [35]. Nasal mupirocin represents the best antimicrobial agent used in decolonization, with an efficacy rate in 91% of treated patients [36]. This decolonization strategy was the most used in the protocols presented by the studies we analyzed, just as the nasal swab was the most implemented colonization research strategy. Our meta-analysis highlighted how the screening and decolonization protocol statistically and significantly reduced total periprosthetic infections and those caused by MSSA and MRSA. This can be explained by the fact that the nasal cavities represent an important reservoir of this pathogen, a site from which it can spread to other areas of the skin surface and, therefore, contaminate the surgical wound during the operating procedure [37]. It has been shown that approximately 80% of the strains causing a staphylococcal infection at the site of surgery have molecular identity with *S. aureus* isolates in the nostrils of affected patients [38]. The data from this meta-analysis support decolonization programs for patients positive for *S. aureus* before undergoing prosthetic implant surgery, as they demonstrated a statistically significant reduction in periprosthetic infections. 

Decolonization with mupirocin is safe for patients and at a low cost for healthcare systems, but some concerns are raised in terms of the risk of developing residencies with this active ingredient. In the literature, the development of resistance has been demonstrated following prolonged administration as an ointment on the skin, while there is no evidence of the development of resistance following administration for short periods, as is in the case of decolonization protocols [39]. Of no less importance are the medical-legal consequences that can be drawn from this topic. PJIs cause serious deterioration in the quality of life, mortality, socioeconomic costs, and also insurance-legal disputes for compensation for iatrogenic damage to the person. In the medical-forensic field, it is still very often believed today that a periprosthetic infection is attributable to errors by the treating surgeons and/or to structural, hygienic, organizational, and technological defects of the hospital structure [40]. Current scientific clinical research confirms the need for a preoperative assessment of the patient’s degree of fragility and requires, as far as possible, the implementation of all conduct and procedures aimed at resolving or attenuating the risk factors to the patient. The search for these risk factors and the consequent procedures implemented to limit their effects are indicators of a medical activity carried out with skill and diligence; on the other hand, the absence of clear evidence on how these precautionary methods have been implemented is qualified as a behavioral criticality of the qualifying doctor. In the indication for a prosthesis, these risks must be carefully weighed, and the probability of negative results must be much lower than that of the positive effects of the prosthetic intervention in the specific person to be treated. Standards of care in medical practice are subject to evolution based on available scientific evidence. Therefore, in light of the evidence in the literature and the results of this meta-analysis, the decolonization of *S. aureus* to reduce the risk of periprosthetic infection could be considered a procedure to follow to maintain an adequate level of care and infection prevention [41]. Failure to adopt evidence-supported protocols could constitute a violation of professional standards, with possible medico-legal implications in the event of postoperative infections. This meta-analysis presents some limitations, including the type of studies included, as it would have been preferable to include randomized controlled clinical trials, and it was not possible to separate the data relating to hip or knee replacements. In addition, in some studies, patients who screened positive for MRSA also received vancomycin as standard perioperative antibiotic prophylaxis, so it was not excluded that the infection rate could be caused by vancomycin use. Furthermore, some differences were observed in the decolonization protocols implemented in the various studies, which could influence the outcomes analyzed. Despite these weaknesses, the work presents several characteristics that make it important, in particular, the high number of subjects analyzed, the number of studies included, the number of results analyzed, and factors that allow us to state how the screening and decolonization of the *S. aureus* is a safe and beneficial practice for the patient and the community. Further studies will need to be conducted to explore the extent to which these types of screening and decolonization protocols actually contribute in socio-economic terms.

## 5. Conclusions

This study showed that the screening and decolonization of *S. aureus* in patients undergoing THA or THA procedures reduced the risk of PJI overall, PJI caused by *S. aureus*, and PJI caused by MRSA, and highlights no difference between the two groups in the onset of PJI caused by other bacteria. The protocol of screening decolonization of this kind of patient represents an important procedure for the safety of the patient and in social-economic and medico-legal terms.

## Figures and Tables

**Table 1:** Inclusion and exclusion criteria applied in the selection of articles.

Inclusion Criteria	Exclusion Criteria
Articles comparing the rate of PJI in patients undergoing elective total hip or knee arthroplasty before/after *S. aureus* screening and/or decolonization protocol. Full text available Comparative studies or randomized controlled trials (RCTs) Studies conducted on humans Studies written in English.	Complete manuscript not available Preclinical studies or ex vivo studies Systematic literature reviews, meta-analyses, commentary, case series Articles written in other languages.

**Table 2:** Characteristics of the articles included in the study. (N.R. = missing data; PJI = periprosthetic joint infection).

Authors	Publ. Year	Group	N° of Patients	Methods of *S. aureus* Screening	Decolonization	PJI Overall	PJI *S. aureus* +	PJI NOT *S. aureus* +
Jeans et al. [22]	2018	Screening	318	Nasal and groin swabs	Octenisan body wash + intranasal Mupirocin for 5 days prior to and after surgery Octenisan body wash	131	23	108
		Control	3593			69	28	41
Baratz et al. [18]	2015	Screening	3434	Nasal swabs	Intranasal mupirocin + chlorhexidine body wash for 5 days	24	13	11
		Control	3080			36	21	15
Hacek et al. [19]	2008	Screening	912	Nasal swabs	Intranasal mupirocin x2 for 5 days	11	7	4
		Control	583			14	10	4
Hadley et al. [20]	2010	Screening	1644	Nasal swabs	Intranasal mupirocin x1 for 5 days	21	3	18
		Control	414			6	1	5
Hofmann et al. [21]	2016	Screening	538	Nasal swabs	Intranasal mupirocin x2 for 5 days + intraoperative betadine irrigation of the wound	6	1	5
		Control	496			15	6	9
Malcolm et al. [23]	2016	Screening	2219	Nasal swabs	Intranasal mupirocin and clorexidine body wash	10	1	9
		Control	1751			17	1	16
Romero-Palacios et al. [24]	2019	Screening	1883	Nasal and throat swabs	Intranasal mupirocin x2 for 5 days and chlorhexidine wipes for 4 days prior to surgery	7	1	6
		Control	8505			42	29	13
Pelfort et al. [25]	2019	Screening	403	Nasal swabs	intranasal mupirocin x2 for 5 days and chlorhexidine gluconate wipes for 5 days prior to surgery	5	1	9
		Control	400			17	8	4
Rao et al. [26]	2008	Screening	636	Nasal swabs	Intranasal mupirocin x2 for 5 days and chlorhexidine gluconate wipes for 4 days prior to surgery	9	0	9
		Control	1330			20	7	8
Rao et al. [27]	2011	Screening	1440	Nasal swabs	intranasal mupirocin x2 for 5 days and chlorhexidine gluconate wipes for 4 days prior to surgery date	17	0	17
		Control	3025			20	4	8
Sankar et al. [28]	2005	Screening	164	Nasal swabs	Intranasal mupirocin or povidone iodine or triclosan	0	0	0
		Control	231			1	1	0
Sporer et al. [29]	2016	Screening	9791	Nasal swabs	Intranasal mupirocin x2 for 5 days and chlorhexidine gluconate wipes for 4 days prior to surgery date	32	N.R	N.R
		Control	1140			13	N.R	N.R

“+” means “ positivity”.

## Data Availability

No new data were created for this study.
